# Performance Degradation in Proton Exchange Membrane Fuel Cell Under Large Load Variation at Rapid Startup: Phenomena and Solution Based on Different Flow Fields

**DOI:** 10.1002/advs.202513666

**Published:** 2025-09-18

**Authors:** Yadong Wang, Fengyang Cai, Zhengkai Tu, Siew Hwa Chan

**Affiliations:** ^1^ School of Energy and Power Engineering Huazhong University of Science and Technology Wuhan 430074 China; ^2^ Energy Research Institute Nanyang Technological University 50 Nanyang Avenue Singapore 637553 Singapore

**Keywords:** Degradation characteristics, Flow field designs, Large load variation, Proton exchange membrane fuel cell, Rapid startup

## Abstract

The durability of proton exchange membrane fuel cells (PEMFCs) under large load variation at rapid startup is critical for commercialization. Experimental and numerical results indicate that the five‐channel serpentine flow field (FSFF) design exhibited the optimal large load variation capability at rapid startup and more effective performance degradation mitigation. Therefore, experimental investigations are conducted on the performance degradation and membrane electrode assembly deterioration of PEMFCs with parallel flow field (PFF) and FSFF designs under rapid dynamic loads (20,000 cycles, 2 s load transients to 3000 mA cm^−2^). The results demonstrate that the multi‐channel FSFF with better gas distribution uniformity and water removal capability significantly mitigates voltage degradation (9.11% vs 20.77% for PFF), reducing both cathode charge transfer resistance and electrochemical surface area degradation. Spatial degradation analysis reveals severe catalyst layer (CL) thinning in outlet regions for both configurations, but FSFF exhibits better degradation mitigation: cathode CL thickness reduction in the outlet region is lower than PFF, accompanied by reduced Pt particle size evolution and agglomeration. These findings highlight the critical role of flow field design in optimizing dynamic durability under large load variation at rapid startup.

## Graphical abstract

This study investigates different flow field designs to optimize the performance and durability of proton exchange membrane fuel cells under large load variation at rapid startup. Specifically, the five‐channel serpentine flow field enhances gas distribution uniformity and water removal capability, thereby improving the large load variation capacity during rapid startup and alleviating the degradation of the membrane electrode assembly.

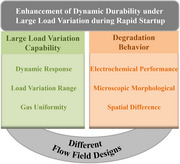

## 1 Introduction

Hydrogen energy, as a core energy carrier for achieving carbon neutrality, is driving profound transformations in the global energy structure through its high energy density, zero carbon emissions, and seamless integration with renewable energy systems.^[^ ^1^ ^]^ Within this context, proton exchange membrane fuel cells (PEMFCs) have emerged as a strategic technology for transportation power and distributed power generation due to their low‐temperature rapid startup capability, high energy conversion efficiency, and zero‐pollution emissions.^[^ ^2^ ^]^

In practical operation scenarios, PEMFCs face a variety of dynamically changing load demands. Among them, the condition of large load variations, where the load current jumps from zero to the working current instantaneously, is particularly challenging. This situation is frequently observed in scenarios like the frequent start‐stop of electric vehicles, distributed generation systems responding to sudden power demand fluctuations, and the instantaneous startup of emergency backup power sources.^[^ ^3^ ^]^ This imposes extreme stress on internal water/thermal distribution, reactant transport, and catalyst stability.^[^ ^4^ ^]^ Rapid load cycling induces significant temperature and humidity gradients across the entire membrane electrode assembly (MEA), particularly in high current density regions.^[^ ^5^ ^]^ High current densities may further lead to reaction gas mass transport limitations, anode dehydration, cathode flooding, excessive heat generation, and non‐uniform distribution, resulting in performance degradation.^[^ ^6^ ^]^ However, current research on the dynamic response under rapid loading conditions has predominantly concentrated at moderate to low current densities, with limited investigation into large load variation operating conditions. Different degrees of voltage undershoots occur with load variations under different operating parameters,^[^ ^7^ ^]^ and the PEMFC could not be successfully loaded at startup when the current was instantaneously changed from 0 to 0.8 A cm^−^^2^. In comparison, it could be successfully loaded at a step current density of 1.5 A cm^−^^2^ after activation.^[^ ^8^ ^]^ Notably, the innovative design of reconstructing cooling channels to establish in‐plane temperature gradients can effectively achieve the synergistic optimization of water management, gas distribution, and thermal management, thereby significantly enhancing the dynamic response capability of PEMFCs under high current density operation.^[^ ^9^ ^]^

Cyclic large load variations represent one of the critical factors contributing to the performance degradation of PEMFCs, with their core impact manifested in the gradual degradation of the cathode catalyst layer (CL), which fundamentally dictates the service lifetime of PEMFCs.^[^ ^10^ ^]^ Operational durability requirements for PEMFC systems typically demand maintaining functionality under cyclic voltage variations between 0.5–0.9 V, while enduring over 300000 dynamic cycles.^[^ ^11^ ^]^ Variable load demands have been demonstrated to cause severe damage to membranes and cathode catalysts, including membrane delamination, catalyst agglomeration, and Pt band formation in regions of high local current density.^[^ ^12^ ^]^ Under high loading rates, transient reactant starvation exacerbates the degradation of the cathode CL, with the severity of damage correlating with reactant starvation gradients.^[^ ^13^ ^]^ Despite extensive research into the mechanisms underlying Pt catalyst degradation and membrane failure under cyclic loading, the specific degradation mechanisms of the cathode CL under large load variations remain insufficiently clarified.^[^ ^14^ ^]^

Flow field design plays a crucial role in addressing the challenges of rapid loading.^[^ ^15^ ^]^ Current common flow field designs in fuel cells primarily include parallel flow field (PFF) and serpentine flow field (SFF). The PFF, characterized by straight channels and low pressure drop, suffers from poor water removal efficiency, leading to cathode flooding and oxygen starvation at high current densities.^[^ ^16^ ^]^ Conversely, the SFF enhances convective transport through tortuous channels but is prone to outlet‐region water accumulation due to extended flow path lengths and gravity‐driven liquid pooling.^[^ ^17^ ^]^ Multi‐channel SFF effectively addresses water management deficiencies of single serpentine configurations by shortening flow path length through distributed channel design, thereby exhibiting superior performance under dynamic conditions.^[^ ^18^ ^]^ Innovative integrated gas diffusion layers (GDLs) designs with wave channels and micro‐tunneled ribs have been proven to achieve faster and gentler mass transfer as well as excellent water management capabilities,^[^ ^19^ ^]^ and an innovative alternating design flow field can effectively enhance the gas concentration in the CL, thereby avoiding the oxygen starvation under high current densities.^[^ ^20^ ^]^

Despite advancements in research on the dynamic performance of PEMFCs, existing studies have primarily focused on conventional load variations. However, investigations into the performance of different flow fields under rapid startup conditions with extremely high current densities, particularly the resulting degradation behavior of the cathode CL, remain limited. This paper conducts a series of comprehensive and in‐depth experimental studies to accurately determine their large load variation capability at rapid startup with different flow field designs, and proposes a rapid dynamic loading protocol involving rapid current loading from zero to a high current density of 3000 mA cm^−2^ within an extremely short duration (2 s). The electrochemical performance, microstructure of the CL, and material degradation patterns of PEMFCs with PFF and the five‐channel serpentine flow field (FSFF) configurations were systematically compared under these rapid dynamic operating conditions. The results provide an experimental benchmark for enhancing the durability of PEMFCs in rapid dynamic environments.

## 2 Experiments

### 2.1 Experimental Setup

**Figure 1:**
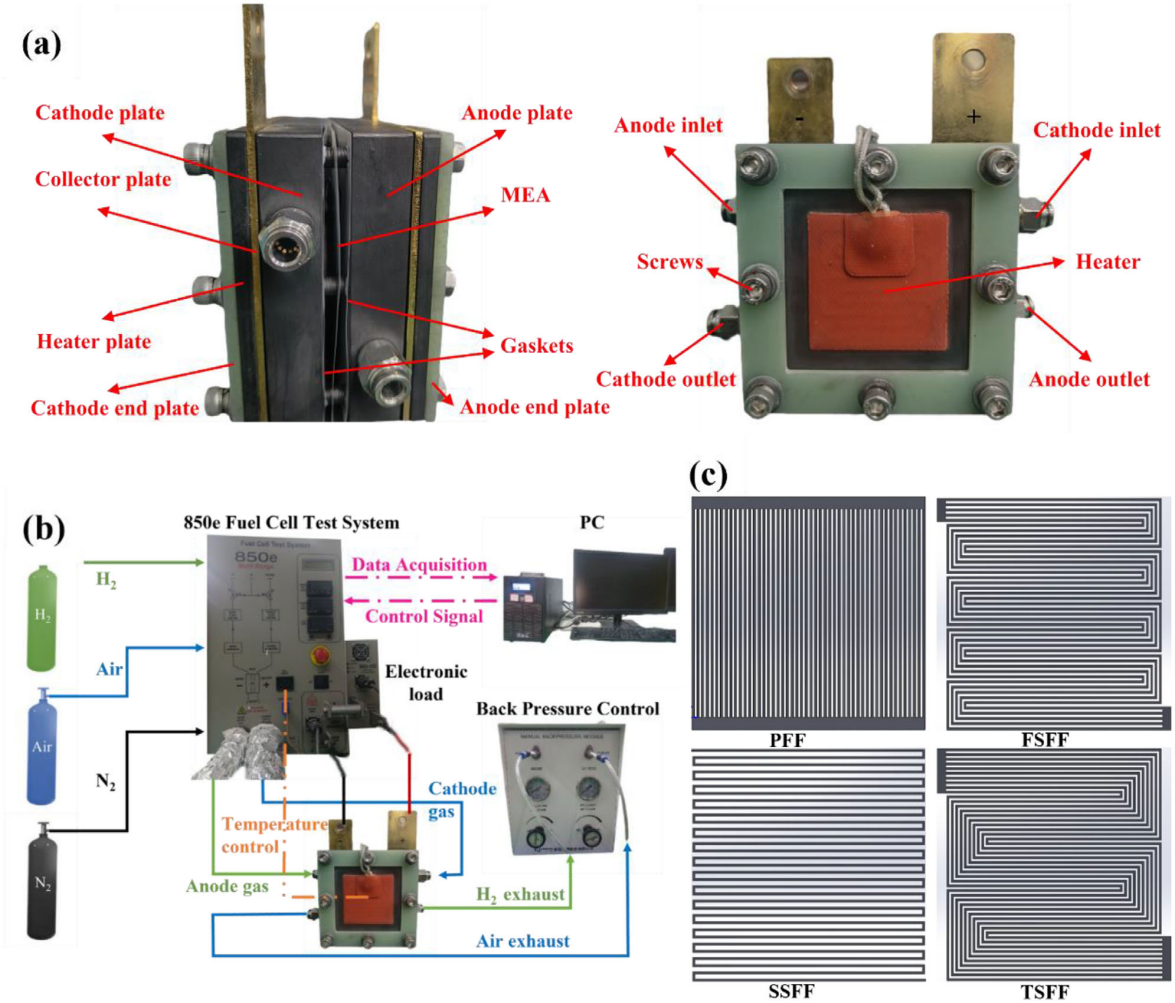
Schematic diagram of experimental setup: a) The structure of the PEMFC. b) The fuel cell test system. c) Different types of flow fields were used in the experiment.

The single PEMFC used in the experiment consisted of bipolar plates, MEA, and heaters. **Figure** **1**a shows the structure of the single PEMFC. The experimental study employed a fuel cell test system (850e), which could accurately evaluate the performance of PEMFCs. Figure 1b presents the test system.

The MEA with an effective active area of 25 cm^2^ (5 cm × 5 cm) was purchased from WUT New Energy Company. The MEA was composed of anode and cathode CL, anode and cathode GDL, and a proton exchange membrane (PEM) prepared by catalyst coated membrane technology. The platinum loadings of anode CL and cathode CL were 0.1 and 0.4 mg cm^−2^, respectively, and both GDLs used polytetrafluoroethylene treated carbon paper. Different types of flow fields were applied to the bipolar plates of the cathode and anode, including PFF, FSFF, single‐channel serpentine flow field (SSFF), and Ten‐channel serpentine flow field (TSFF). The width of the channel was set to 0.5 mm, the depth to 1 mm, and the rib width to 0.5 mm (the rib width of SSFF was 1 mm). The size of the flow field was the same as that of the membrane electrodes, which was 25 cm^2^, as shown in Figure 1c. The two bipolar plates and the MEA were clamped and fixed with 8 screws, and the assembly force was 2.94 N·m to avoid adverse effects caused by uneven stress distribution. Heaters were installed on both sides of the bipolar plates to control the temperature of the single cell.

### 2.2 Experimental Scheme

Following assembly, the PEMFC underwent a gas‐tightness test to ensure experimental safety and data reliability. Before the experiment, a single hydrogen‐air PEMFC was first activated. During the activation process, the current density was gradually increased from 0 to 80 A with an increment of 1 A each time. After the voltage had stabilized, the next load was applied. The current density of 80 A was continuously maintained for 2 h to ensure stability, and then the current density was gradually decreased from 80 A to 0, with a decrement of 2.5 A each time. This process was repeated three times to complete the entire activation process. The initial performance characterized after this conditioning was considered the reference for subsequent cyclic loading experiments. The operating parameters for this study are detailed in Table 1.

**Table 1:** Operating parameters.

Parameter	Value
Anode Gas	H_2_(99.99% purity)
Cathode Gas	Synthetic air (79% N_2_, 21% O_2_)
Anode/Cathode Back Pressure (Pa)	20000
Operating Temperature (°C)	60
Anode/Cathode Relative Humidity	100%
Anode (H_2_) Stoichiometric Ratio	1.5
Cathode (Air) Stoichiometric Ratio	2.5

In this experiment, the limit current that could be reached under different flow field designs from zero to high current density was investigated. The performance degradation at rapid startup was evaluated by analyzing this value. The limit voltage of the PEMFC was set to 0.25 V. That is, when the voltage undershot below 0.25 V, the working condition is considered unfeasible, and the PEMFC is shut down. Each set of load variation currents undergoes at least ten repeated tests. Only when all test results meet the preset criteria is the set of conditions deemed qualified. On this basis, three sets of data are randomly selected for plotting and presentation.

To investigate the durability of PEMFCs under rapid operating conditions, this study designed a dynamic loading protocol involving rapid current loading from 0 to 3000 mA cm^−2^ within an ultra‐short duration of 2 s. Cyclic durability tests were conducted separately for PEMFCs equipped with PFF and FSFF configurations. To comprehensively assess the impact of rapid loading on cell performance and component degradation, polarization curves, cyclic voltammetry (CV), and electrochemical impedance spectroscopy (EIS) were performed at intervals of 5000 cycles using the 850e test system. The specific operating conditions are summarized in Table 2.

**Table 2:** Operating Condition Step.

Step	Condition	Duration
1	Cell activation and initial performance test	8 h
2	Load transient from 0 to 3000 mA cm^−2^	2 s
3	Rated current density operation	5 s
4	Gradual load reduction to 0	2 s
5	Zero current operation	5 s
6	Repeat steps 2–5 for 5000 cycles	
7	Performance characterization (polarization curves, EIS, CV)	
8	Repeat steps 2–7 for 4 cycles (total 20000 load cycles)	
9	System shutdown and post‐test analysis	

The post‐test analysis employed scanning electron microscopy (SEM, JEOL JSM‐IT800) to examine the cross‐sectional morphology of the MEA, transmission electron microscopy (TEM, FEI Tecnai G2 20) to characterize Pt particle size distribution in CLs, and X‐ray photoelectron spectroscopy (XPS, Kratos AXIS‐ULTRA DLD‐600 W) to analyze Pt valence states. These complementary techniques enabled the systematic evaluation of MEA degradation mechanisms under rapid load cycling for both flow field designs.

## 3 Results and Discussion

### 3.1 The Large Load Variation Response

**Figure 2:**
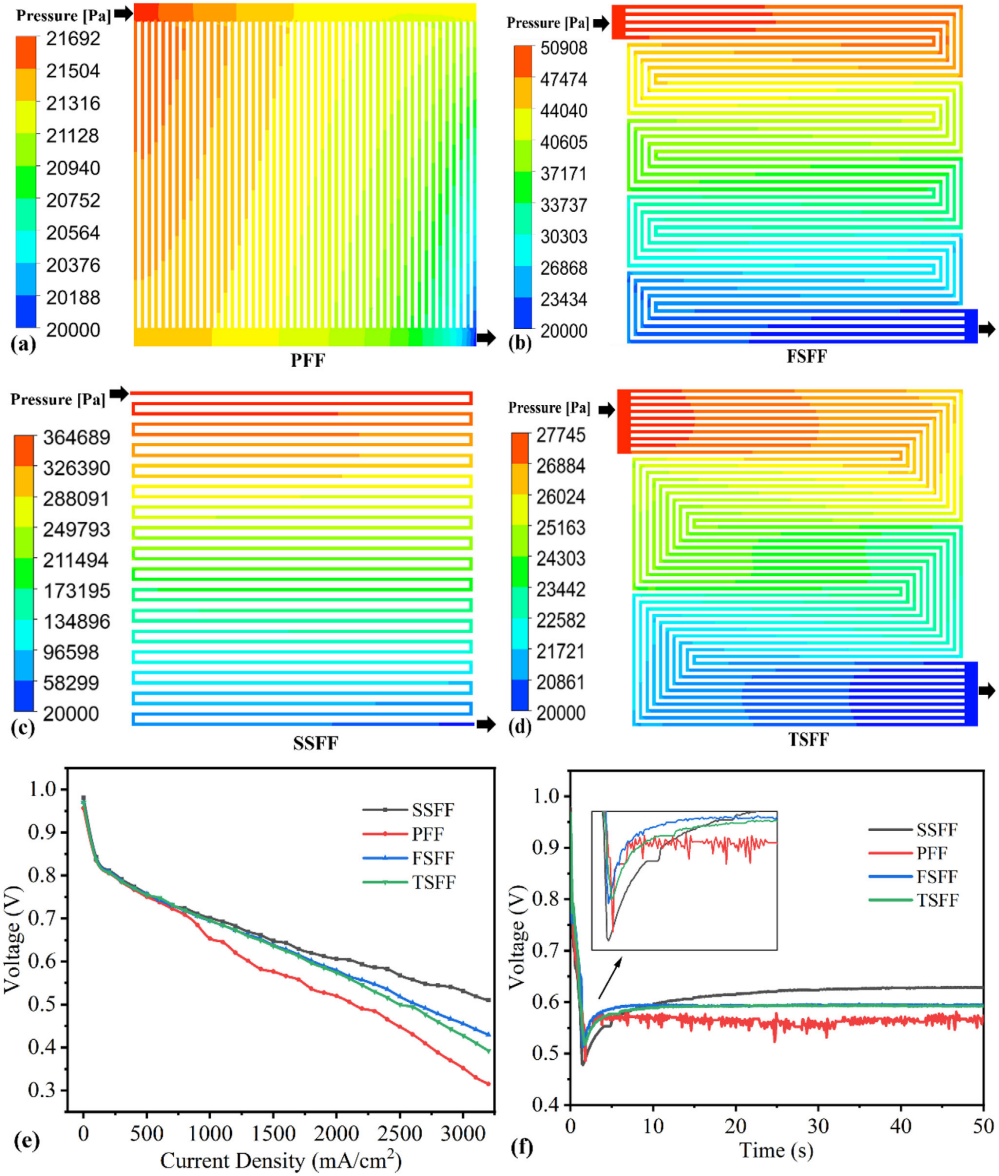
Static pressure distributions of different flow fields: a) PFF. b) FSFF. c) SSFF. d) TSFF. e) Schematic diagram of polarization curves at different flow fields. f) Schematic comparison of voltage response for PEMFCs with different flow fields applying 0–50 A variable load current.

Different flow field designs have varying gas distribution and water management capabilities. When the inlet mass flow rates remain constant, **Figure** **2**a–d illustrates the static pressure distributions of different flow fields in the numerical simulations. For all serpentine flow fields except the PFF, static pressure decreases gradually from the inlet to the outlet of the flow channels. The SFF exhibits the largest pressure drop of 344689 Pa, followed by the FSFF at 30908 Pa and the TSFF at 7745 Pa. Notably, the pressure drop across the inlet and outlet increases progressively with channel length. In contrast, the PFF shows a uniform gradual pressure decline from inlet to outlet, with minimal pressure drop within each channel, and the total inlet‐outlet pressure drop is only 1692.3 Pa. The higher pressure drops in serpentine flow fields are beneficial for liquid water removal, enhancing gas distribution uniformity and mitigating flooding in the GDL.^[^ ^21^ ^]^

The performance difference of fuel cells assembled with different flow channel structures is substantial. Figure 2e shows the polarization curves of the fuel cell using different flow fields. From the figure, the SSFF exhibits the best performance due to its larger pressure drop level,^[^ ^22^ ^]^ followed by the FSFF and TSFF, and the PFF had the worst performance. The PFF exhibits inferior operational stability, as demonstrated by pronounced fluctuations in its polarization curve.^[^ ^23^ ^]^ This instability is likely attributed to the misalignment between the horizontal orientation of the inlet/outlet gas flow and the vertical orientation of the parallel channels, inducing significant instability in the gas flow.^[^ ^24^ ^]^

To clearly compare the large load variation voltage response phenomena at rapid startup under different flow field types, Figure 2f shows the dynamic response during a 2 s loading time at 0–50 A. From the figure, the SSFF has the largest voltage undershoot, followed by the PFF, the TSFF, and the FSFF. The time to return to a steady state is in ascending order: the SSFF, the TSFF, the FSFF, and the PFF. The SSFF shows the highest steady‐state voltage, the TSFF and FSFF are close to each other, and the PFF has the lowest steady‐state voltage with poor stability. The SSFF has the largest voltage undershoot and the longest time to recover the steady state, indicating poor variable load capability. Therefore, it is not suitable for application scenarios with frequent and large load variations. In contrast, the PFF has the shortest time to recover stability after a variable load, but its steady‐state operation stability is poorer.^[^ ^18^ ^]^ By comparison, the FSFF has better steady‐state and dynamic capabilities, and its large load variation capability is also superior.

**Figure 3:**
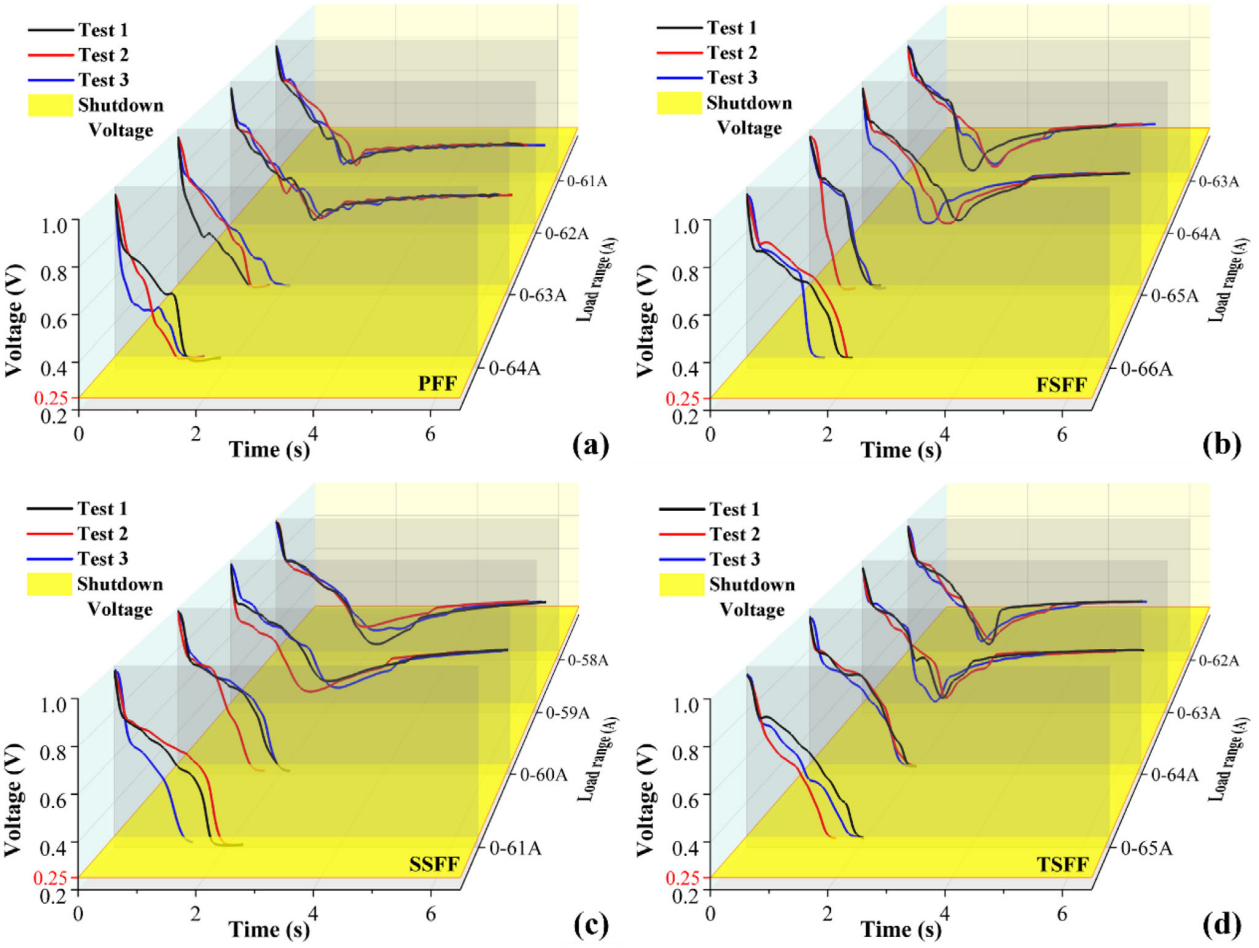
The results of the PEMFC large load variation test using different flow fields with a temperature of 60 °C, a humidity of 100%, and a loading time of 2 s: a) PFF. b) FSFF. c) SSFF. d) TSFF.

To explore the maximum attainable load variation range during rapid start‐up, experiments were conducted at 60 °C, 100% relative humidity, and a 2 s loading time, with sequential testing of each flow field design. The PEMFC was loaded to a predetermined current density in a short period, and multiple experiments were carried out to avoid random experimental errors. **Figure** **3**a–d shows the dynamic response of PEMFCs with different flow fields. Observing the PFF, it can be observed that when the load currents were 0–61 and 0–62 A, the voltage started to rise gradually after a significant undershoot, with minimum voltages remaining above the 0.25 V protection, indicating successful loading. Thus, it can be inferred that the loading current below 0–62 A can be achieved. When the load currents were 0–63 and 0–64 A, the voltage dropped sharply below the limiting voltage of 0.25 V, indicating loading failure. Based on the experimental results, the maximum attainable load variation range during rapid startup of the PFF is initially evaluated to be 0–62 A under the operating conditions of an operating temperature of 60 °C, a relative humidity of 100%, and a loading time of 2 s.

By comparing the maximum attainable load variation ranges of PEMFC with different flow fields, it was found that the FSFF can achieve 0–64 A, followed by the TSFF (0–63 A) and the PFF at (0–62 A), while SSFF can only load 0–59 A. The SSFF demonstrates superior steady‐state performance due to its large pressure drop,^[^ ^17b^ ^]^ yet exhibits the poorest large load variation capacity. This performance arises from its elongated flow path, which induces localized reactant starvation and liquid water flooding. Insufficient gas supply and impaired water removal under dynamic conditions degrade its transient response, limiting its ability to sustain higher current demands.^[^ ^25^ ^]^ The FSFF and TSFF have a balance of advantages, making their large load variation performance slightly higher than that of both the PFF and SSFF. However, the performance between flow field types remains minimal under such rapid loading conditions, suggesting limited potential for improving the maximum attainable load variation range during rapid startup through flow field optimization alone.

### 3.2 Electrochemical Characterization After Cycles Loading

Previous studies have demonstrated that the FSFF exhibits superior large load variation performance compared to other configurations, while the PFF is most widely adopted in commercial applications due to its low flow resistance. Thus, this study conducts a comprehensive durability assessment of these two configurations under rapid load variations.

**Figure 4:**
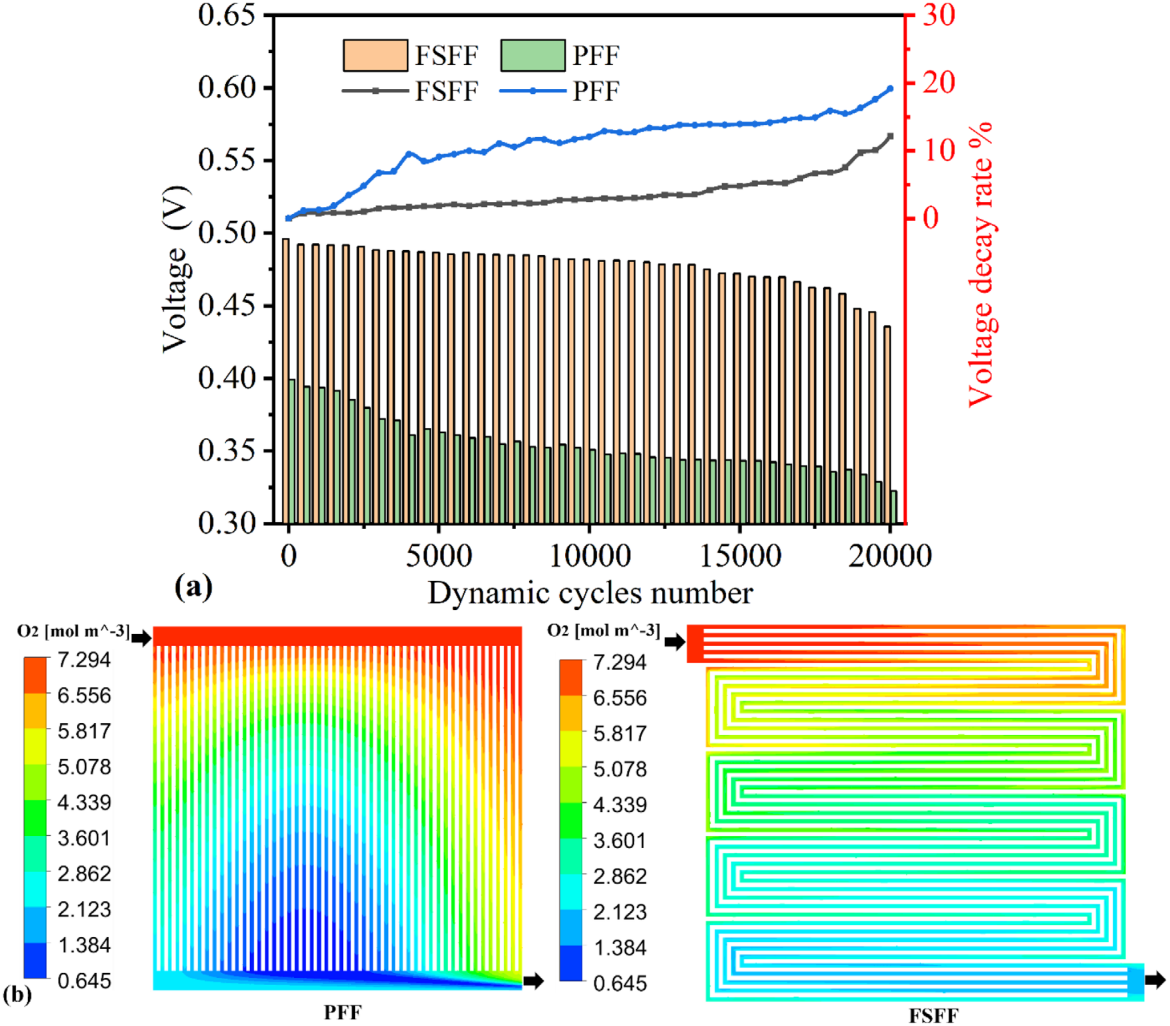
a) The trend chart of the voltage at the reference current density point (3000 mA cm^−2^) over time during the durability operation. b) The oxygen molar concentration distributions of different flow fields.

The average voltage response at the reference current density of 3000 mA cm^−2^ was recorded every 500 cycles during the rapid dynamic loading tests, and its temporal evolution is depicted in **Figure** **4**a. Both PFF and FSFF configurations exhibited significant voltage degradation at high current densities. Specifically, PFF experienced a larger voltage degradation rate, decreasing from an initial 0.40 to 0.32 V (19.16% degradation), compared to FSFF's 0.50 to 0.44 V decline (12.14% degradation). PFF demonstrated a “rapid‐slow‐rapid” pattern. In contrast, FSFF showed a trend with gradual degradation acceleration toward the end of testing. This discrepancy may arise from PFF's poor gas distribution uniformity and inferior water management capability, leading to early‐stage liquid water accumulation under high‐frequency transients, causing localized gas starvation and rapid voltage degradation. In the intermediate phase, persistent flooding in the flow channels led to a gradual degradation rate. Finally, accelerated degradation in the late phase was likely driven by irreversible component deterioration. Conversely, FSFF's superior drainage efficiency mitigated transient flooding, resulting in stable degradation rates until late‐stage component fatigue caused accelerated decline.

Specifically, Figure 4b illustrates the poor oxygen distribution uniformity in PFF, where the central region showed significantly lower oxygen concentration than the side regions, accompanied by a distinct hypoxic zone. This phenomenon was primarily attributed to insufficient gas supply caused by the excessively low flow velocity in this area. Such a deficiency renders the PFF more susceptible to water accumulation and subsequent flooding, thereby impeding oxygen transport efficiency.^[^ ^26^ ^]^ In contrast, the complex geometry of FSFF promoted enhanced mixing and more homogeneous gas distribution, effectively mitigating the degradation‐inducing inhomogeneities. Additionally, the higher pressure drop in FSFF is beneficial for liquid water removal and mitigating flooding in the GDL.^[^ ^21^ ^]^ These numerical results not only validated the experimental observations but also provided mechanistic insights into the degradation behaviors of the two flow field designs.

**Figure 5:**
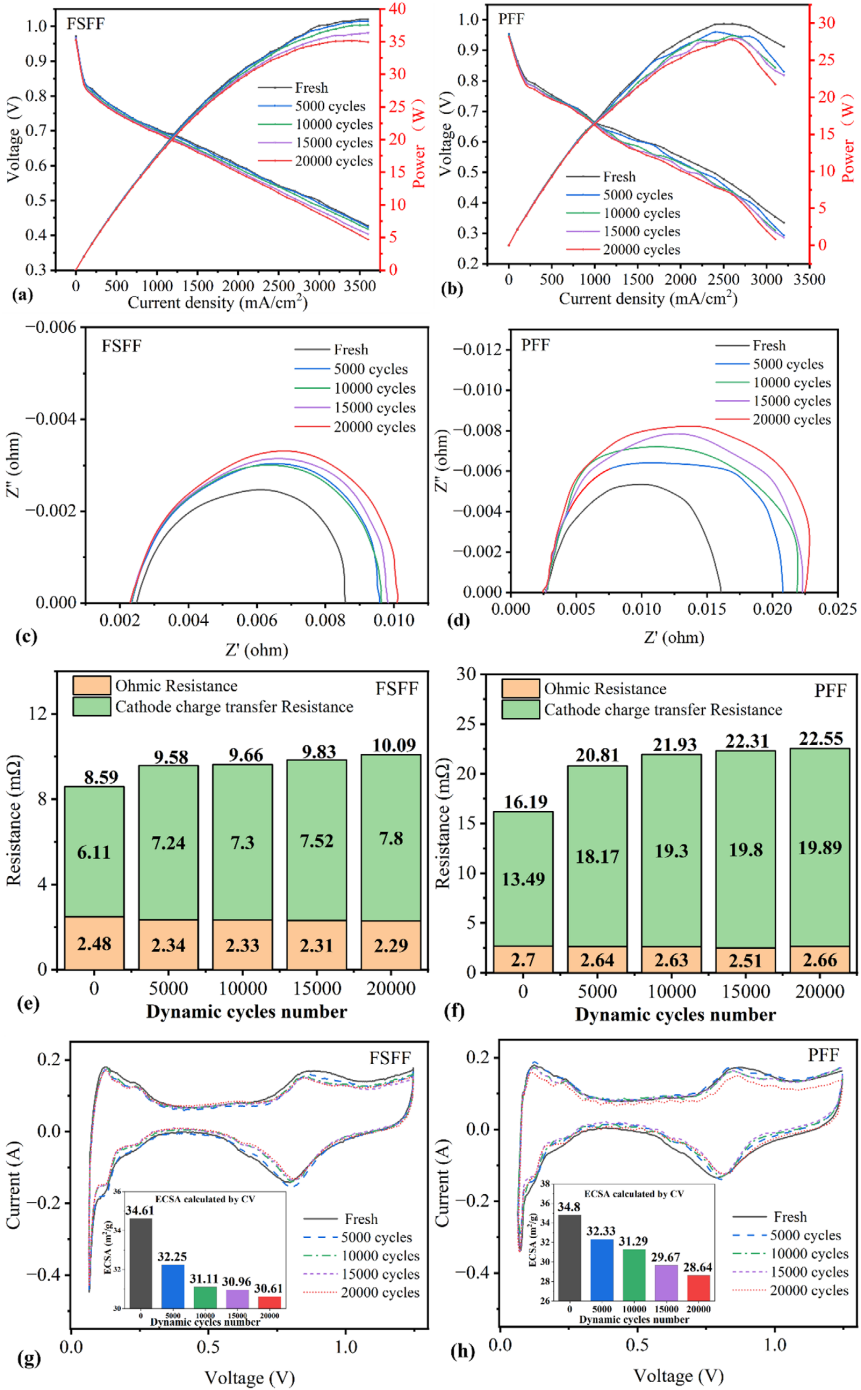
The performance degradation of PEMFC for different operating times: a,b) The polarization and power curves of FSFF and PFF. c,d) The EIS curves. e,f) The comparison of resistance. g,h) The CV curves.

The evolution of polarization curve performance is presented in **Figure** **5**a,b. FSFF exhibits smooth polarization curves with stable voltage outputs, whereas PFF demonstrates significant voltage fluctuations. This discrepancy likely arises from PFF's vertical channel orientation conflicting with horizontal gas inlet/outlet flows, causing unstable gas distribution. In contrast, FSFF's predominantly horizontal channels align with gas flow directions, ensuring stable transport. Both PFF and FSFF show declining polarization curves and power outputs with increasing cycle numbers. Notably, voltage degradation rates at low current densities are comparable between configurations, but PFF experiences significantly greater degradation at high current densities (3000 mA cm^−2^). This is attributed to FSFF's higher pressure drop, reducing liquid water accumulation under rapid water generation conditions. Notably, the voltage degradation rate of the PFF at 3000 mA cm^−2^ during polarization testing (20.77%) exceeds its cyclic average degradation rate (19.16%), confirming that its poor gas distribution uniformity and rapid loading conditions synergistically exacerbate irreversible degradation. Conversely, the FSFF exhibits a lower voltage degradation rate during polarization testing (9.11%) compared to cyclic operation (12.14%). The primary reason may be attributed to the rapid load changes during cyclic loading and the short running time at the reference current point. Under such circumstances, the internal water and thermal states of the PEMFC are difficult to reach equilibrium within a short period, and liquid water may temporarily flood the CL and GDL. This leads to significant voltage fluctuations, resulting in partial reversible degradation during the operation of working conditions. In contrast, during the polarization curve test, each operating point runs stably for a long time under steady operating conditions, which allows these reversible degradations to be largely recovered.

As shown in Figure 5c–f, the diameter of the semicircle in EIS spectra represents the charge transfer resistance (Rct) of the cathode oxygen reduction reaction, while the first intersection with the abscissa corresponds to ohmic resistance (R_Ω_). Results indicate that both PFF and FSFF exhibit increasing Rct values with load cycle progression. Specifically, FSFF's cathode Rct increased from 6.11 to 7.8 mΩ (27.66% growth), whereas PFF's Rct rose from 13.49 to 19.89 mΩ (47.44% growth), significantly exceeding FSFF. This discrepancy highlights more severe degradation within PFF's cathode CL compared to FSFF. Conversely, neither configuration showed significant R_Ω_ growth, confirming that the CL degradation dominates performance degradation under rapid load cycling.^[^ ^13a^ ^]^

CV analysis reveals a progressive reduction in the charge area of hydrogen desorption peaks for both PFF and FSFF configurations as the number of dynamic loading cycles increases, as shown in Figure 5g,h. The electrochemical surface area (ECSA), calculated by integrating the hydrogen desorption peak, serves as a critical indicator of catalyst activity.^[^ ^27^ ^]^ For the FSFF, the ECSA decreased from an initial value of 34.61 m^2^ g^−1^ to 32.25, 31.11, 30.96, and 30.61 m^2^ g^−1^ after successive cycles, representing a total reduction of 11.56%. In contrast, the PFF exhibited a more pronounced ECSA decline from 34.80 m^2^ g^−1^ to 32.33, 31.29, 29.67, and 28.64 m^2^ g^−1^, corresponding to a total loss of 17.72%. The observed ECSA degradation is attributed to Pt particle dissolution, migration, agglomeration, and redeposition within the CL.^[^ ^28^ ^]^ The rapidly short loading time (2 s) and high current density (3000 mA cm^−2^) exacerbate gas starvation and flooding, accelerating these degradation mechanisms.^[^ ^29^ ^]^ Notably, the FSFF's superior drainage capability mitigates liquid water accumulation ^[^ ^30^ ^]^ thereby reducing Pt degradation rates compared to the PFF.

### 3.3 Morphological Characterizations after Cycle Loading

**Figure 6:**
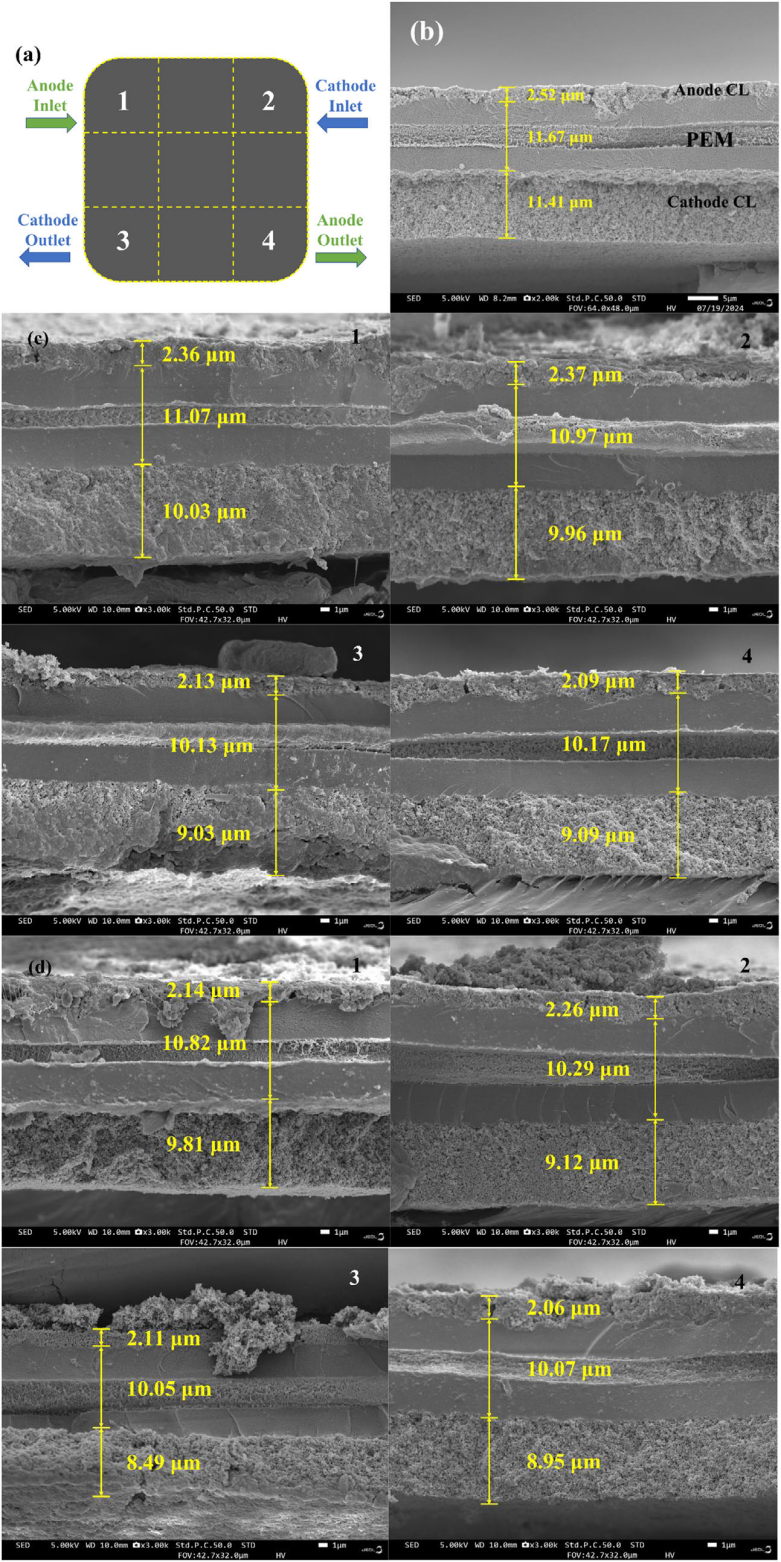
a) Schematic diagram of cathode MEA sub‐area division. b) The cross‐sectional morphology image of fresh MEA. The cross‐sectional images and thickness profiles from different regions after cycle loading: c) FSFF. d) PFF.

Following cyclic dynamic loading tests, the MEA was divided into nine regions as illustrated in **Figure** **6**a, with regions 1–4 corresponding to the anode inlet, cathode inlet, cathode outlet, and anode outlet, respectively. The cross‐sectional morphology and thickness distribution of these regions were analyzed via SEM before and after 20000 rapid loading cycles. Figure 6b displays the fresh MEA structure, while Figure 6c,d presents cross‐sectional images and thickness profiles for the PFF and FSFF configurations after cycle loading. The three distinct layers in the images represent the anode CL, PEM, and cathode CL.

**Figure 7:**
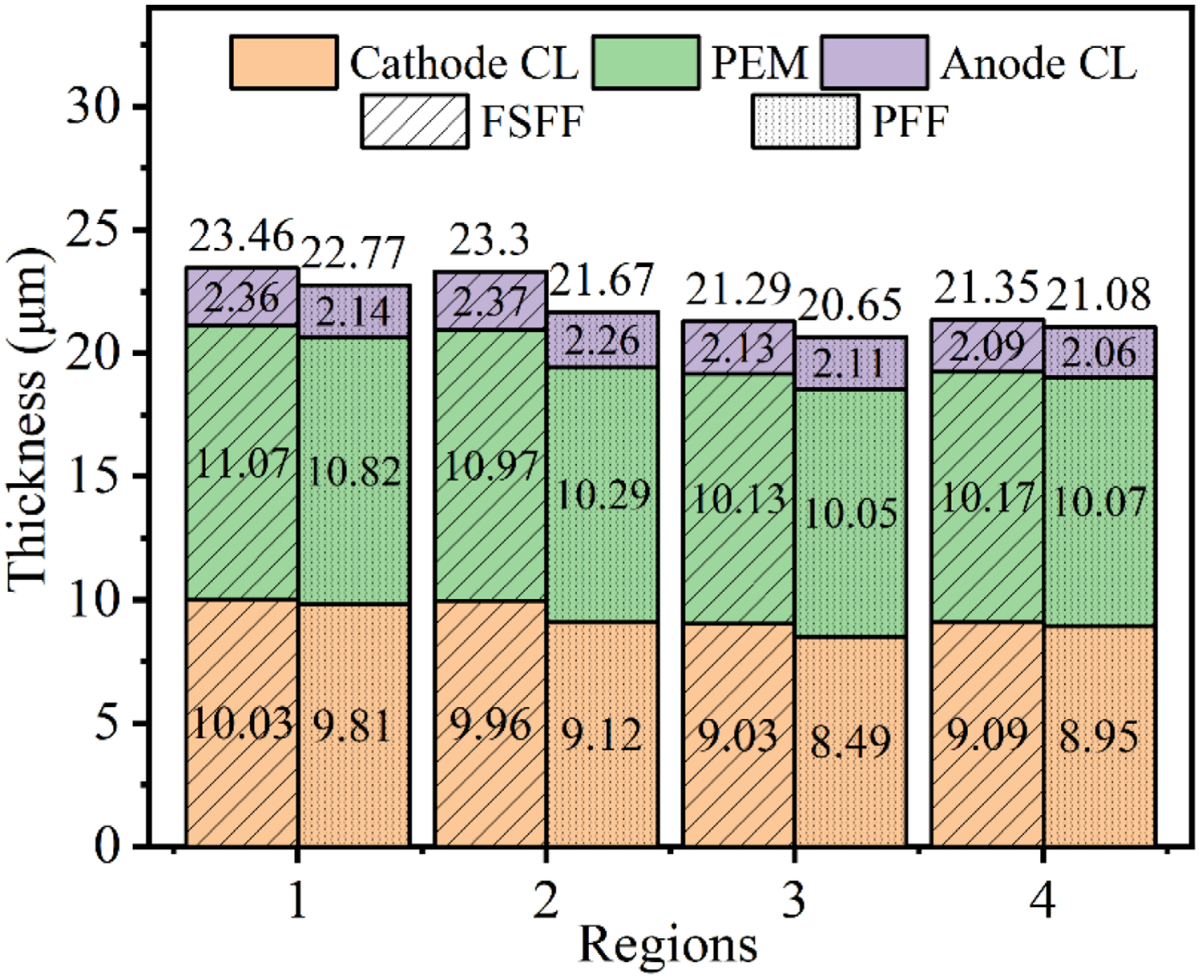
The thickness of each component of the MEA in different regions.

Significant thickness reductions were observed in both anode/cathode CLs and PEM across all regions, with distinct degradation gradients. For PFF, cathode CL degradation was most severe at regions 3 (25.59%) and 4 (21.56%), compared to regions 1 (14.02%) and 2 (20.07%). This likely results from gas starvation and gravity‐induced liquid water accumulation at outlets, exacerbating CL degradation and carbon corrosion.^[^ ^31^ ^]^ Anode CL thickness reductions followed 15.08%, 10.32%, 16.27%, and 18.25% respectively, with the most severe degradation at the anode outlet due to hydrogen starvation caused by cathode‐to‐anode water diffusion under transient conditions.^[^ ^32^ ^]^ PEM thinning was less pronounced (7.28%、11.83%、13.88%、13.71% for regions 1–4, respectively), attributed to mechanical fatigue from cyclic humidity variations. The thickness variation of each component of the MEA in different regions is shown in **Figure** **7**.

FSFF demonstrated lower degradation rates in all regions due to greater gas distribution uniformity and water management capability. Cathode CL thickness reductions were 12.09%, 12.71%, 20.86%, and 20.33%, while anode CL reductions were 6.35%, 5.95%, 15.48%, and 17.06%. PEM degradation rates (5.14%, 5.99%, 13.20%, 12.85%) were also mitigated. Similarly, these levels of degradation are all more severe in the outlet regions. The CL degradation led to component delamination, increased electron transfer resistance, and performance decline.

The TEM was employed to investigate the Pt particle morphology and size distribution in cathode CL of fresh and cycled MEAs. Fresh MEA samples exhibited uniform Pt particle dispersion with an average diameter of 4.62 nm, as shown in **Figure** **8**a. Both FSFF and PFF configurations showed significant Pt agglomeration after rapid load cycling, particularly pronounced in outlet regions 3 and 4, as shown in Figure 8b,c.

Figure 8TEM images and the frequency distribution histograms of the cathode Pt catalyst with overlaid statistical annotations (mean ± standard deviation). a) Fresh. b) FSFF. c) PFF.

**Figure:**
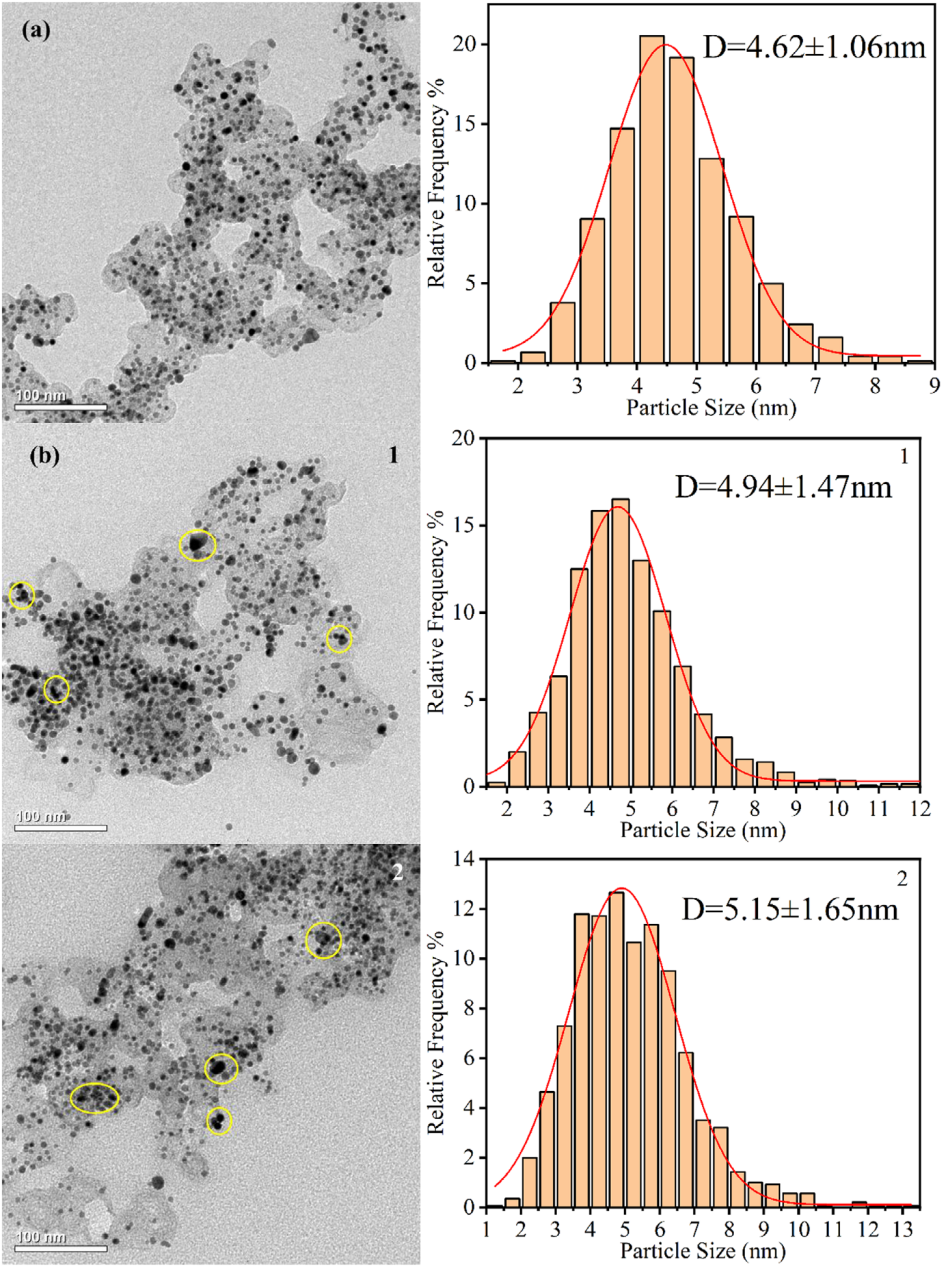

**Figure:**
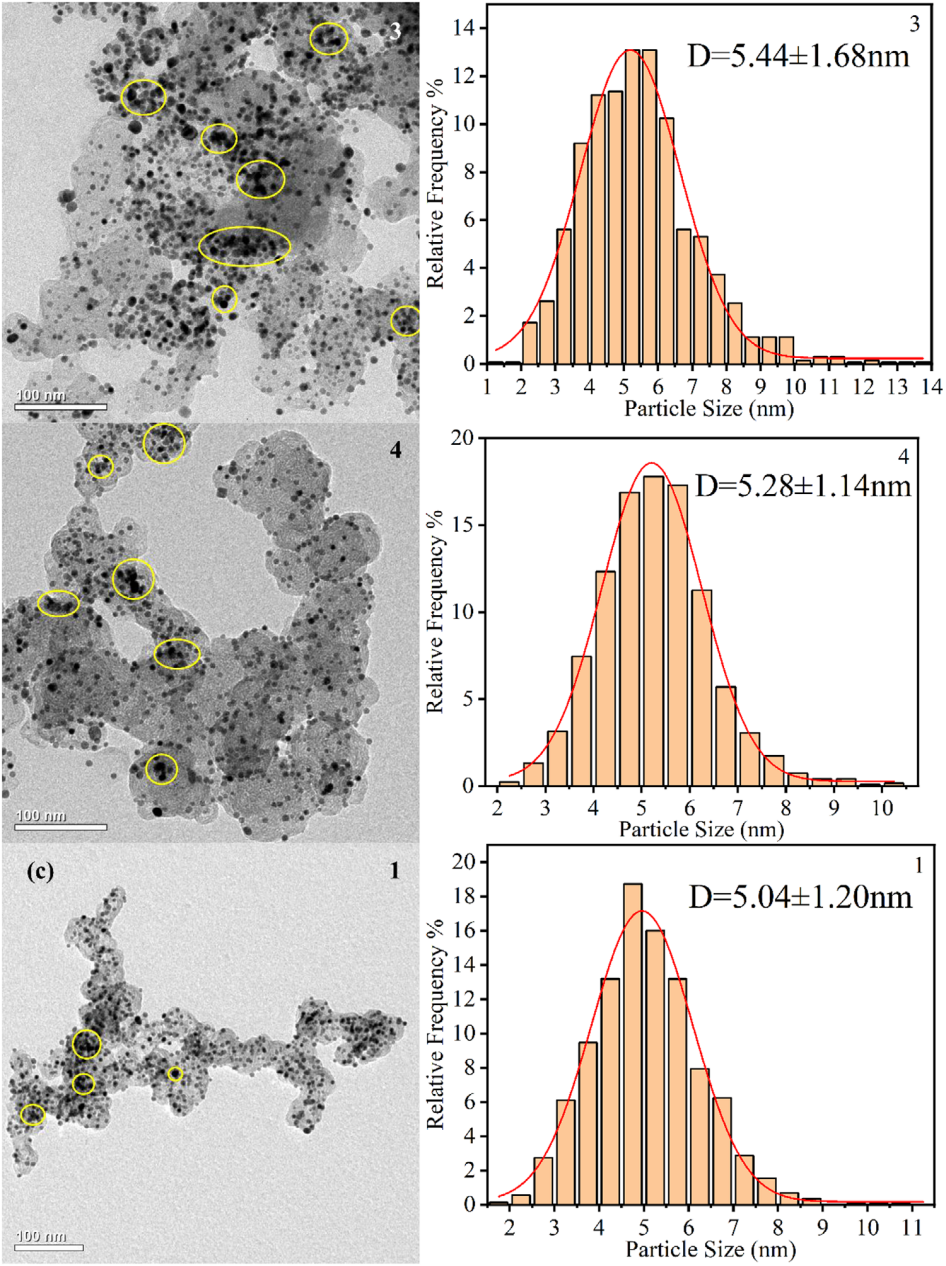

**Figure:**
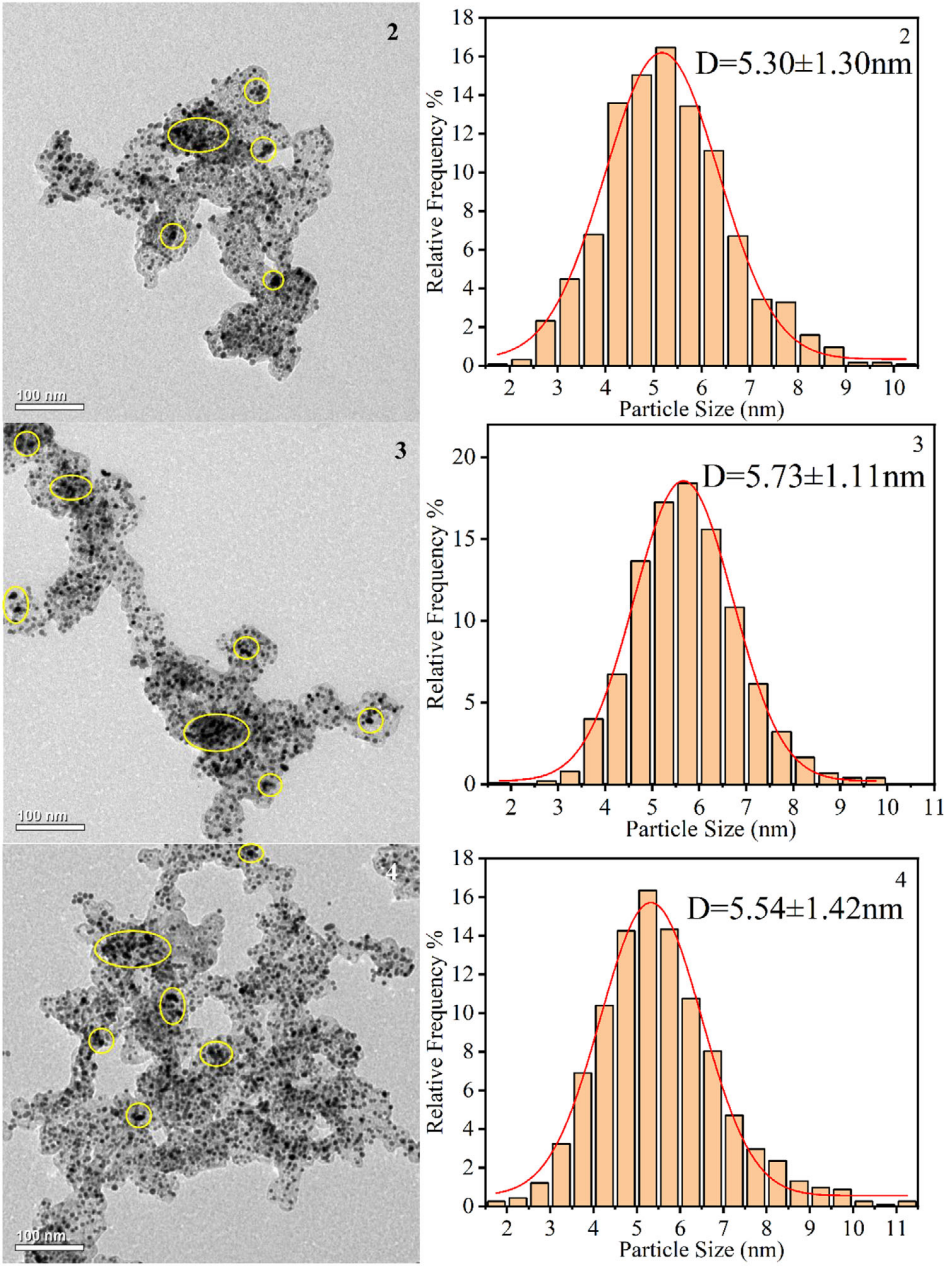

This morphological evolution is attributed to the Ostwald ripening mechanism, where liquid water accumulation induced localized high potentials, causing Pt^2^⁺ dissolution from smaller particles and redeposition onto larger ones. This process increased average Pt particle size and reduced ECSA, directly correlating with performance degradation.^[^ ^33^ ^]^ For PFF, Pt particle size increases were most severe at region 3 (24.03%), followed by region 4 (19.91%), region 2 (14.72%), and region 1 (9.09%). The elevated proportion of large Pt particles in outlet regions corroborated SEM observations of accelerated CL degradation in these areas.

FSFF configurations demonstrated reduced Pt agglomeration rates, with average particle size increases of 17.74% (region 3), 14.29% (region 4), 11.47% (region 2), and 6.93% (region 1). This mitigation effect is attributed to FSFF's high pressure drop and superior water management, which minimized liquid water retention and delayed Ostwald ripening. These results highlight the critical role of flow field design in maintaining Pt particle stability under transient conditions.

**Figure 9:**
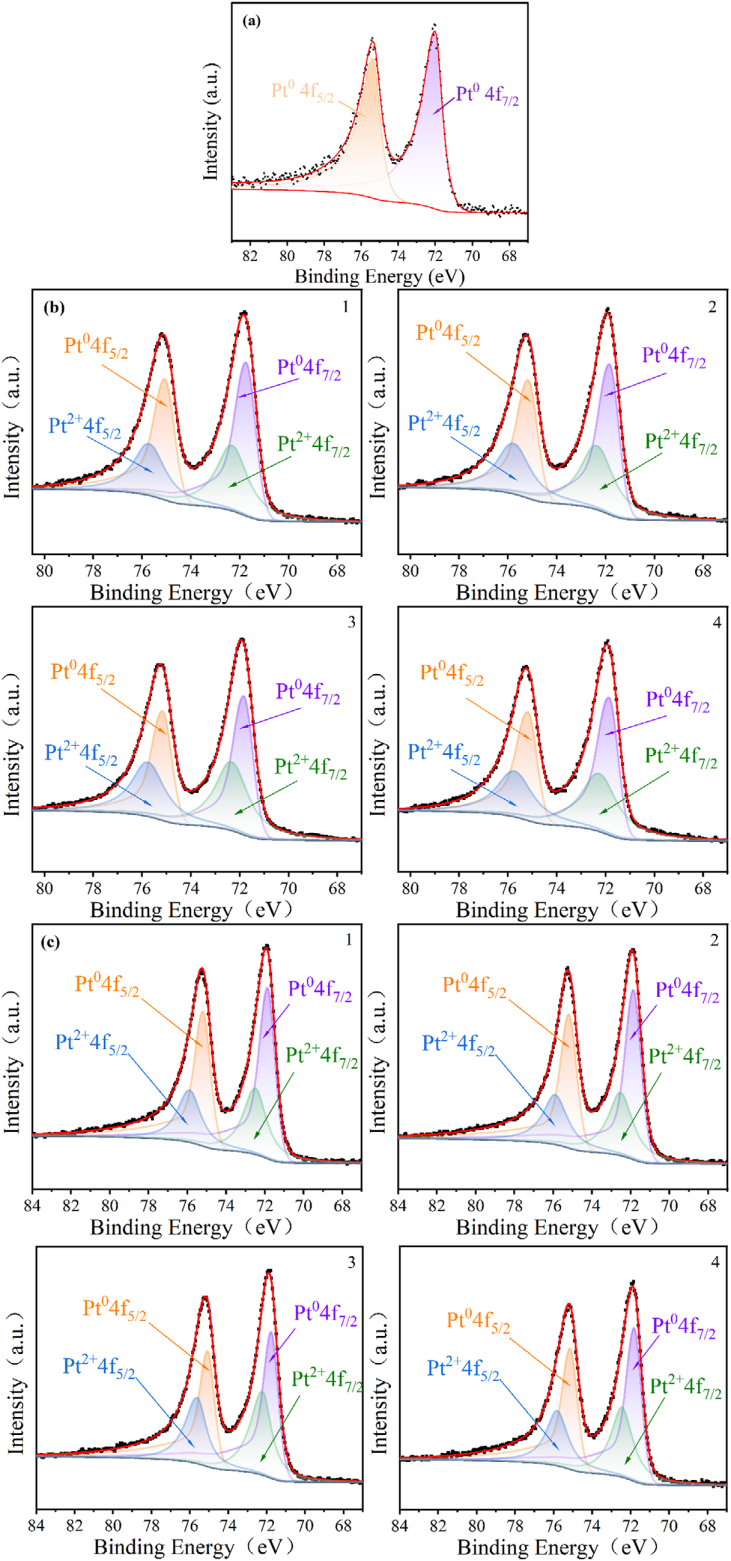
a) The Pt‐4f XPS mapping of fresh MEA. The Pt‐4f XPS mapping from different regions after cycles loading. b) FSFF. c) PFF.

To characterize the valence states of Pt in the CL, XPS was employed to analyze the Pt chemical states across different regions of the cathode in both fresh and cycled MEAs. For the fresh MEA, the Pt‐4f XPS spectra exhibited two distinct peaks at binding energies of 72.05 and 75.35 eV, corresponding to the Pt⁰ 4f_7_/_2_ and Pt⁰ 4f_5_/_2_ orbitals, respectively, confirming that Pt predominantly existed in the metallic state (Pt⁰) initially, as shown in **Figure** **9**a.

Following 20000 rapid dynamic loading cycles, significant changes in Pt oxidation states were observed. The spectra revealed two additional peaks at 72.35 and 75.75 eV, assigned to Pt^2+^ (PtO), indicating partial oxidation of Pt during operation, as shown in Figure 9b,c.

Table 3 summarizes the relative atomic percentages of Pt with different oxidation states in the cathode CL of MEAs across distinct regions for PFF and FSFF configurations. The oxygen reduction reaction activity of Pt particles varies significantly depending on their oxidation states, with metallic Pt(0) exhibiting the highest catalytic activity.^[^ ^34^ ^]^ Increased formation of Pt oxides (Pt^2+^) reduces kinetic performance and accelerates catalyst dissolution, migration, and agglomeration. After the test, it was revealed that substantial oxidation of Pt(0) to Pt(II) after rapid dynamic load cycling, with approximately 40% of metallic Pt oxidized in severely affected regions, leading to marked catalytic performance degradation. Notably, the PEMFC operated with FSFF exhibited slightly higher atomic percentages of Pt^2+^ in corresponding regions compared to PFF. This discrepancy may be attributed to the higher operating voltages of FSFF, which generate more localized high‐potential regions, thereby promoting Pt oxidation.^[^ ^35^ ^]^ Furthermore, regions 3 and 4 displayed elevated relative Pt^2+^ content, indicating intensified Pt oxidation near the outlet regions, which exacerbates CL degradation. These findings align with the MEA degradation patterns observed in SEM and TEM morphological analyses, confirming the spatial correlation between Pt oxidation severity and structural deterioration.

**Table 3:** Relative Atomic Percentages of Pt Valence States in Cathode CLs.

Region	Pt (0)		Pt (II)	
	PFF	FSFF	PFF	FSFF
Anode inlet (1)	66.28%	61.50%	33.72%	38.50%
Cathode inlet (2)	65.23%	58.92%	34.77%	41.08%
Cathode outlet (3)	60.37%	53.27%	39.63%	46.73%
Anode outlet (4)	62.69%	56.83%	37.31%	43.17%

## 4 Conclusion

This study compares the response capabilities of PEMFCs with different flow field designs under large load variations at rapid startup, and examines the degradation of PFF and FSFF configurations under rapid dynamic load cycling, where current density rapidly loads to 3000 mA cm^−2^ within 2 s through 20000 operational cycles. Key findings are summarized as follows:

1. During rapid startup under large load variations, FSFF demonstrates the smallest voltage undershoot, with a recovery time to steady state second only to PFF, and achieves the largest attainable load variation range. Thus, a comprehensive evaluation confirms that FSFF exhibits the optimal large load variation capability during rapid startup.
2. Under rapid dynamic loading (3000 mA cm^−2^), PFF exhibited significantly higher degradation rates compared to FSFF, as evidenced by voltage degradation (20.77% vs 9.11%), cathode charge transfer resistance increase (47.44% vs 27.66%), and ECSA reduction (17.72% vs 11.56%). Morphological characterization revealed severe thinning of the PFF's CL, leading to Pt particle growth and agglomeration, whereas FSFF mitigated CL degradation owing to greater gas distribution uniformity and water management capability. Notably, both configurations exhibited substantial Pt oxidation during cyclic operation.
3. FSFF has a higher pressure drop due to its complex channel geometry, which improves gas uniformity and eliminates the central hypoxic zone caused by slow flow in PFF. The high‐pressure drop of the FSFF effectively enhances water removal efficiency but also increases parasitic power consumption. In practical applications, striking a balance between these factors is critical.
4. Region‐specific degradation patterns were observed due to gas distribution inhomogeneity and liquid water accumulation. The degradation at the bottom of the PEMFC, i.e., gas outlets, is more severe than at the top. This is evidenced by variations in CL thickness, average catalyst particle size, and the relative content of Pt (II) species. Moreover, the spatial difference in degradation becomes more pronounced under PFF compared to FSFF.

These findings underscore the critical role of flow field design in enhancing PEMFC durability under dynamic operating conditions. Future efforts should prioritize advanced flow field optimization and catalyst material innovation to improve resilience against rapid load cycling.

## Conflict of Interest

The authors declare no conflict of interest.

## Author Contributions

Y.W. contributed to writing the original draft. F.C. was responsible for methodology. Z.T. provided supervision, and S.H.C. contributed resources.

## Data Availability

The data that support the findings of this study are available on request from the corresponding author. The data are not publicly available due to privacy or ethical restrictions.
